# Evolutionary characterization and pathogenicity of the highly virulent human-porcine reassortant G9P[23] porcine rotavirus HB05 strain in several Chinese provinces

**DOI:** 10.3389/fmicb.2025.1539905

**Published:** 2025-03-14

**Authors:** Xi Li, Jingjing Wang, Yuankui Zhang, Yarong Zhao, Yanli Shi

**Affiliations:** Beijing Biomedicine Technology Center, Zhaofeng Hua Biotechnology (Nanjing) Co., LTD, Beijing, China

**Keywords:** porcine rotavirus, G9P[23], reassortment, evolutionary characterization, pathogenicity

## Abstract

Rotavirus A (RVA), a member of the *Sedoreoviridae* family, is significant intestinal pathogen that cause diarrhea in both piglets and humans. During of an outbreak that struck nursing piglets with diarrhea, a human-porcine reassortment rotavirus, named as RVA/Pig-wt/China/HB05/2023/G9P[23] (hereafter referred to as HB05), was identified. This specific strain was found to be prevalent in pig farms in several regions, including Hebei, Liaoning, Sichuan, Zhejiang and Henan, and caused significant economic losses from March to August 2023. To further explore the evolutionary diversity of HB05, a comprehensive analysis of all gene segments was conducted. The genome constellation was identified as G9-P[23]-I5-R1-C1-M1-A8-N1-T1-E1-H1. Nucleotide sequence identity and phylogenetic analyses indicated that the NSP3 gene of HB05 is most closely related to the corresponding genes of Human strains, with the highest homology at 95.45% identity. The other genes (VP1-VP4, VP6-VP7, NSP1-NSP2, NSP4-NSP5) exhibited the closest relationship to porcine strains, with the highest homology ranging from 94.79 to 98.89% similarity. Therefore, it is likely that HB05 originated from genetic reassortment between porcine and human rotaviruses. The pathogenicity study performed on 3-day-old piglets revealed that severe diarrhea manifested 8 h post-infection after oral inoculation with the PoRV HB05 strain at a dose of 2 × 10^5.5 TCID50/mL per piglet. To our knowledge, this marks the first report of a prevalent and highly virulent human-porcine reassortment G9P[23] rotavirus A (RVA) strain identified in mainland China. This finding provides valuable insights into the evolutionary traits of the G9P[23] strain and suggests a possible risk of cross-species transmission.

## 1 Introduction

Species A rotaviruses (previously designated as Group A rotaviruses), members of the *Sedoreoviridae* family, are a major cause of acute diarrhea in humans and various animal species (Matthijnssens et al., 2022; Crawford et al., 2017). The viral genome is represented by double-stranded RNA and consist of 11 gene segments that encode for six structural proteins (VP1–VP4, VP6, and VP7) and six nonstructural proteins (NSP1–NSP6) (Crawford et al., 2017). Based on antigenic differences in the structural protein VP6, rotaviruses can be further classified into nine distinct serogroups, ranging from A to J (RVA to RVJ) species A-J, excluding rotavirus E (Mihalov-Kovács et al., 2015; Bányai et al., 2017; Vlasova et al., 2017). Among these serogroups, A, B, C, E, and H have shown the capacity to infect pigs. Within the population of RVs affecting pigs, RVA is consistently recognized as the most prevalent species, with an occurrence rate reaching up to 67.3% (Vlasova et al., 2017). In human cases of RVs infection, species A rotavirus is the most prevalent cause of childhood infections and accounts for a staggering 90% of all infections (LeClair and McConnell, 2023; Gomez-Rial et al., 2020). The sequences of the two outer capsid proteins of rotaviruses, namely the glycosylated VP7 and the protease-sensitive VP4, are used to categorize the viruses into distinct G and P genotypes, respectively (Matthijnssens et al., 2008). In China, G9P[23] is the most prevalent genotype found in pigs (Li et al., 2022). Rotavirus A (RVA) strains are regarded as potential zoonotic pathogens. To date, in humans, at least ten G genotypes (G1–G5, G9–G12, and G26) and seven P genotypes (P[4], P[6], P[8], P[13], P[14], P[19], and P[25]) of porcine origin have been identified (Vlasova et al., 2017). However, certain genotype combinations, including G9P[23], have been circulating among humans, pigs, and dogs (Chen et al., 2019; Komoto et al., 2017; Yan et al., 2019). Our objective was to enhance our understanding of the rotavirus strains prevalent during the post-vaccine-introduction period in China, specifically focusing on the triple attenuated vaccine (PEDV-TGEV-PoRVA) for diarrhea (Tang et al., 2024). The Rotavirus Classification Working Group (RCWG) established an RV genome system for genotyping in 2008, which is based on 11 gene segments. This nomenclature method employs a notation such as Gx-P[x]-Ix-Rx-Cx-Mx-Ax-Nx-Tx-Ex-Hx to determine the genotype of a specific species A rotavirus strains. Each capital letter corresponds to VP7-VP4-VP6-VP1-VP2-VP3-NSP1-NSP2-NSP3-NSP4-NSP5 gene segments respectively, while ‘x’ represents unique genotypes associated with that particular strain (Matthijnssens et al., 2008). Such terminology plays a pivotal role in providing valuable insights into the involvement of RV in reassortment events and cross-species infections. Porcine rotavirus (PoRV) exhibits genetic diversity within the swine population globally (Genz et al., 2023; Hull et al., 2020; Park et al., 2022). Furthermore, the distribution and prevalence of specific genotypes can exhibit regional and temporal variations. Moreover, genetic variations within the same genotype contribute significantly to the overall genetic diversity of the virus. Understanding these variations is crucial for the development of vaccines and treatment strategies capable of effectively targeting the diverse array of rotavirus strains prevalent in pig farms.

In this investigation, we have uniquely identified the prevalent occurrence of G9P[23] rotavirus A (RVA) strain on swine farms across multiple regions in China, such as Liaoning, Sichuan, Zhejiang, and Henan. From March to August 2023, this particular strain caused substantial economic losses. We have thoroughly characterized the genome of this strain and investigated its origins. The epidemiological implications of these findings are significant, indicating a potential for interspecies transmission and the emergence of novel strains that could pose a public health threat. In this study, the HB05 strain was found to be widespread in pig farms in several regions, including Hebei, Liaoning, Sichuan, Zhejiang, and Henan, causing significant economic damages from March to August 2023. The neutralizing epitope regions of the HB05 VP7 and VP4 proteins were compared with those of several RV strains obtained from GenBank. The analysis revealed a high degree of variability within the G9P[23] strain. To evaluate the pathogenicity of the identified G9P[23] RVAs, experimental infection was performed on 3-day-old piglets, which demonstrated a considerable level of virulence and shedding. The results of this study offer valuable insights into the epidemiology of porcine rotavirus and its potential risks to public health, emphasizing the need for ongoing surveillance and the development of effective control strategies.

## 2 Materials and methods

### 2.1 Ethics statement

The animal experiments were carried out in accordance with the Chinese Laboratory Animal Regulations, the Guidelines for the Care of Laboratory Animals (Ministry of Science and Technology of the People’s Republic of China), and Laboratory Animal Requirements for Environment and Housing Facilities (GB 14925–2010; national Technical Committee for Standardization). This research protocol was approved by the Laboratory Animal Ethical Committee of Kemu Feng Biotechnology (Beijing) Co., LTD under license number KMF20230815-3.

### 2.2 Sampling, examining and virus isolation

Between March and August 2023, a large-scale outbreak of watery diarrhea symptoms in suckling piglets was reported in several Chinese provinces and cities. This was thought to be caused by PEDV (porcine epidemic diarrhea virus) infection. Samples were collected and sent to our testing facility for thorough virus isolation and pathogen diagnosis. The sample locations were Beijing, Liaoning, Henan, Sichuan, Fujian, Hebei, Zhejiang Province and Inner Mongolia Autonomous Region. In these eight regions, 98 fecal samples were collected from randomly selected sick piglets. The samples were subjected to RT-PCR analysis using specific primers which were used for the detection of PoRV, PEDV, PDCoV (porcine deltacoronavirus) and TGEV (transmissible gastroenteritis virus) as indicated in Table 1.

**Table 1:** The detection primers of enterovirus.

Primers name	Primer sequence	Product size (bp)
PoRV-VP7-F	ATGTATGGTATTGAATATACCACAGTT	785
PoRV-VP7-R	TGTATWAYWGCTACRTTYTCYCTTGGTCC	
PDCoV-M-F	GGCAAATTATTGTTTTCATTGCGATCATATGGGCGC	625
PDCoV-M-R	CTTATACAGGCGAGCGTCACCGGCCTTTGAAG	
TGEV-S-F	TCGCAATAATAGTAATGACCTTTAT	480
TGEV-S-R	TTAAACCACCAAAGGTCTACAA	
PEDV-M-F	GCATCCTTATGGCTTGCATCAC	337
PEDV-M-R	GTGCCAGATGAAGCATTGACTGAACG	

One-Step RT-PCR Kit (Nanjing Vazyme, China) was used for RT-PCR amplification after total viral RNA was extracted from the stool samples using Viral RNA Extraction Kit (TianGen, China) according to the manufacturer’s instructions. Samples that tested positive were sent to Beijing Qingke Biotechnology Co. Ltd. for sequencing. The results were then uploaded to the NCBI database for Nucleotide BLAST analysis. The MegAlign program was used to align the sequencing data. According to the NCBI BLAST results, the VP7 gene sequences from Hebei, Liaoning, Sichuan, Zhejiang, and Henan are all 100% homologous and belong to the G9 genotype strain. Following that, MA104 cells were individually inoculated with these VP7 sequence-identical samples. PoRV strains of the G9 genotype were successfully isolated after nine passages that included isolation, adaptation, and plaque purification. The virus titer was then determined in MA104 cells.

### 2.3 Indirect immunofluorescence assay (IFA)

The supernatant of PoRV-infected MA104 cells in 6-well cell culture plates was taken out. The cells were then washed three times with PBS. After that, the cells were fixed with ice-cold ethanol for 30 min at −20°C. Following fixation, cells were blocked with 5% nonfat dry milk in PBS for an hour at room temperature (RT) following three PBS washes. After three times PBS washes, cells were incubated with a polyclonal anti-VP6 antibody (prepared in our laboratory, diluted 1:2000) for 1 h at 37°C. Coralite488-conjugated goat anti-rabbit IgG (H + L), a fluorescent secondary antibody (Proteintech, diluted 1:200), was subsequently incubated for an additional hour at 37°C. Finally, the cells were washed three times before examination under a fluorescence microscope.

### 2.4 Electron microscopic observation

When CPE was observed in more than 80% of cells, PoRV-infected MA104 cells were harvested for PoRV virion particle imaging. Subsequently, 0.45 μm filters were used to filter the clarified viruses. Samples were then centrifuged using a Beckman ultracentrifuge with a 70Ti rotor at 30,000 rpm and 4°C for 1 to 5 h to pellet virus particles. The virus pellets were resuspended in 1 mL of PBS buffer and further purified by a sucrose density gradient (30, 45, and 60%). The purified virus samples were then negatively stained with phosphotungstic acid and examined under an electron microscope.

### 2.5 Full genome sequencing

RNA extracted from the F9 generation strains was subjected to next-generation sequencing (NGS) analysis using the Illumina Novaseq 6000 platform at Beijing Qingke Biotechnology Co., Ltd. This project employed the Illumina sequencing technology to complete the genomic scanning sequencing of the virus strains. An Illumina paired-end (PE) library was constructed, and after performing quality control on the obtained sequencing data, bioinformatics analysis methods were utilized to complete the whole-genome scanning of the virus strains. The obtained clean reads were subjected to *de novo* assembly, that is, de novo sequencing assembly. SPAdes and SOAPdenovo software were used to assemble the next-generation sequencing data. Contigs with a length of ≥1,500 bp were selected for depth statistics.

The G9 genotype porcine rotavirus, isolated from various provinces including Hebei, Liaoning, Sichuan, Zhejiang, and Henan, exhibited 100% gene segment homology based on sequence alignment and nucleotide sequence identity analysis. The NGS results were then submitted to the National Center for Biotechnology Information (NCBI) for further analysis. Individual segment Nucleotide BLAST analysis, nucleotide sequence identity analysis, and sequence alignment identified this strain as the G9P[23] strain, which has been designated as HB05. Genotyping was performed using the method proposed by Gomez-Rial et al. (2020) and Matthijnssens et al. (2008), where the PoRV genotyping method is considered consistent when the percentage of nucleotide sequences in 11 gene fragments exceeds a certain threshold. To further investigate the genetic origins of HB05, phylogenetic trees were constructed using MEGA7 software and the Maximum Likelihood Model (GTR) with bootstrap support (1,000 replication steps).

### 2.6 Pathogenicity experiments

Ten 3-day-old piglets from a farm without signs of diarrhea, all of which tested negative for PoRV, PEDV, TGEV and PDCoV by RT-PCR assays, participated in this study. The sows’ serum showed no evidence of neutralizing antibodies against PoRV, as determined by pre-established methods (Tang et al., 2024). The piglets were divided into two groups: five in the experimental group and five in the control group, each housed separately. The experimental group was administered the PoRV HB05 strain orally (2 mL per piglet containing 1.0 × 10^5.5 TCID50/ml), while the control group received oral vaccination with DMEM (2 mL per piglet). They were given powdered milk at regular intervals of 3 h. Piglets were closely observed daily for clinical signs and viral shedding, with severity of symptoms and diarrhea assessed according to pre-established criteria (Wang et al., 2018). The observation period of the piglets after challenge was 7 days and the surviving piglets were euthanized at the end of the experiment. The results of the study were presented as the mean ± standard deviation of three experiments and the graphs were created using GraphPad Prism 8.0.

## 3 Results

### 3.1 Detection of RVA infection in feces samples

A serious outbreak of acute diarrhea in nursing piglets resulted in high morbidity and mortality in pig farms. The affected piglets showed severe symptoms, including weakened intestinal walls, yellow, watery or semi-solid feces and weight loss. Stool samples were collected from 98 sick piglets randomly selected from eight different regions: Beijing (10 samples), Liaoning (10 samples), Henan (5 samples), Sichuan (10 samples), Fujian (20 samples), Hebei (15 samples), Inner Mongolia (8 samples) and Zhejiang (20 samples). The aim was to determine whether the diarrhea was caused by PEDV or other viral infections such as PoRV, PDCoV or TGEV. For this purpose, the samples were subjected to RT-PCR analysis with specific primers. The results showed that all samples tested positive for RVAs but negative for PEDV, PDCoV and TGEV. According to the sequencing results (Table 2), rotaviruses G4 (3/8) and G5 (5/8) were detected in Inner Mongolia, while Liaoning had genotypes G3 (5/10) and G9 (5/10). In Sichuan, Zhejiang and Henan there were only rotaviruses of genotype G9, in Fujian only rotaviruses of genotype G3, in Hebei both rotaviruses of genotype G5 (5/15) and G9 (10/15) and in Beijing only genotype G5. When analyzing the VP7 gene sequences obtained from samples with the G9 genotype, it became clear that Beijing was the exception as it had 95% sequence identity in the VP7 gene, while the remaining regions had perfect 100% homology.

**Table 2:** Distribution of RVA positive samples.

Source	Total number	Number of pig farms	Number of RVA positive samples	Number of samples of different G genotypes			
				G3	G4	G5	G9
Inner Mongolia	8	3	8	0	3	5	0
Liaoning	10	1	10	5	0	0	5
Henan	5	1	5	0	0	0	5
Sichuan	10	2	10	0	0	0	10
Fujian	20	2	20	20	0	0	0
Hebei	15	2	15	0	0	5	10
Beijing	10	2	10	0	0	3	7
Zhejiang	20	2	20	0	0	0	20
Total number	98	15	98	25	3	13	57

### 3.2 Virus isolation, identification and growth curves of HB05

We attempted to isolate porcine rotavirus (PoRV) from PCR-positive diarrhea samples that did not contain other pathogens commonly associated with diarrhea. The VP7 sequence-consistent G9 rotavirus-positive stool samples from Hebei, Liaoning, Sichuan, Zhejiang and Henan provinces were successfully isolated and purified. High-throughput sequencing of the F9 generation virus demonstrated strain consistency; we referred to it as HB05.

The overall results of virus isolation are summarized as follows: A total of 14 strains were isolated and identified, including two G5 strains and one G4 strain from Inner Mongolia, one G3 strain and one G9 strain from Liaoning, one G9 strain each from Henan and Sichuan, two G3 strains from Fujian, one G9 strain and one G5 strain from Hebei, one G9 strain and one G5 strain from Beijing, and one G9 strain from Zhejiang.

**Figure 1:**
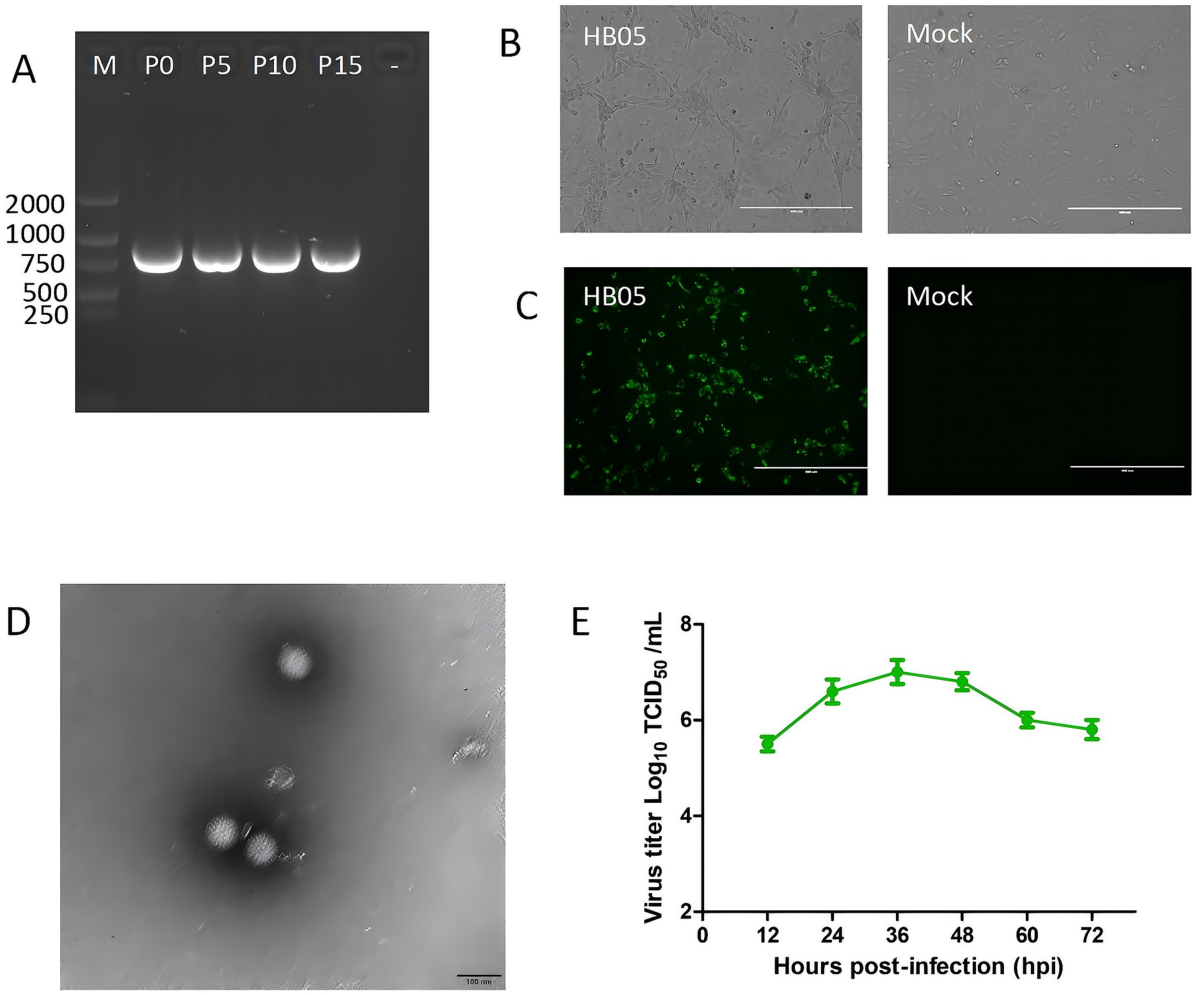
Virus isolation, identification, and growth curves of HB05. **(A)** RT-PCR products using the VP7 specific primer for 0, 5, 10 and 15 consecutive generations of HB05 strain. **(B)** CPE infected with HB05 strain in MA104 cells. Scale bars = 400 mm. **(C)** Immunostaining analysis of VP6 protein expression in MA104 cells after infected with HB05 strain. Scale bars = 400 mm. **(D)** Electron microscopic images of purified PoRV particles. Scale bars = 100 mm. **(E)** Growth kinetics of HB05 in MA104 cells. Data was presented as mean ± SD by triplicates.

After the initial inoculation, the HB05 isolate showed significant cytopathic effects (CPE), consistent with RV infection in MA104 cells. To verify whether the HB05 strain could maintain its viability and replication capacity in MA104 cells, it was continuously propagation for 15 generations. Subsequently, the VP7 genes of the P0, P5, P10, and P15 generations of the virus were identified as positive, as shown in Figure 1A. This finding also indicates that the isolated virus can be stably passaged *in vitro*. After gene amplification and sequencing of VP7, it was observed that the sequences of the VP7 gene remained unaltered throughout the passages. However, it was noted that the virus demonstrated enhanced adaptability to the cells. As a result, when the same inoculation dose was applied during the passage process, the lesion developed more rapidly, and the time required for virus harvesting was shortened.

At 24 h post-infection, a typical cytopathic effect (CPE) was observed during the continuous spread of the virus. The CPE was characterized by cells that had enlarged, spherical, and debris (Figure 1B). Furthermore, the propagation of PoRV strain HB05 in MA104 cells was validated by identifying VP6 polyclonal antibodies directed against the VP6 protein. As shown in Figure 1C, 12 h post-infection, PoRV VP6 protein-specific immunofluorescence was observed in the vast majority of cells, with the VP6 protein localized primarily to the cytoplasm. In contrast, mock MA104 cells exhibited neither CPE nor VP6 staining.

The typical particles of the HB05 virus, which were separated from infected MA104 cells, were examined by transmission electron microscopy (TEM). Negative staining under the electron microscope revealed particles with a wheel-like feature, closely resembling spheres. These particles had an approximate diameter of 70 nm, as shown in Figure 1D. Additionally, the growth kinetics analysis demonstrated that HB05 was highly adaptable and replicated effectively in MA104 cells. At 36 h post-infection, the viral infection titer peaked at 10^7.0 TCID50/mL (Figure 1E).

### 3.3 Genome sequences of HB05

Unbiased high-throughput sequencing was performed using the Illumina Novaseq 6000 platform. A total of 8,840,896 reads were generated for the viral genome, with an average length of approximately 150 base pairs. The complete sequences of 11 gene segments of the sequenced strain were successfully identified. Sequence alignment, Nucleotide BLAST analysis, and nucleotide sequence identity analysis all confirmed that this strain belonged to genotype G9-P[23]-I5-R1-C1-M1-A8-N1-T1-E1-H1 (Table 3). It was found to be identical to previously identified Chinese porcine rotavirus G9P[23] strains, such as SC11, which has the genotype G9-P[23]-I5-R1-C1-M1-A8-N1-T1-E1-H1 (Chen et al., 2019).

**Table 3:** The highest nucleotide sequence identities between HB05 and other known RVA strains.

Gene	Closely related strains	Homology	Genotype	Accession No.
VP7	RVA/Pig-wt/CHN/SCLSHL-2-3/2017/G9P[23]	97.22%	G9	MH137265.1
VP4	RVA/Porcine/CHN/rJXAY01/G5P[23]I12	97.24%	P[23]	PP975113.1
VP6	RVA/pig/CHN/GX/DM4/2022/G5P13I5	98.24%	I5	OQ799772.1
VP1	RVA/Pig/China/SC11/2017/G9P[23]	97.38%	R1	MH624173.1
VP2	RVA/Pig/China/JSJR2023/G4P[23]	97.62%	C1	PP100150.1
VP3	RVA/pig/China/NMTL/2008/G9P[23]	94.79%	M1	JF781160.1
NSP1	RVA/pig/CHN/SD/LYXH2/2022/G4P6I1	96.78%	A8	OQ799883.1
NSP2	RVA/pig/Tanzania/RP019/2019/G4P[6]	96.13%	N1	ON092400.1
NSP3	RVA/Human-wt/NPL/TK1797/2007/G9P[19]	95.45%	T1	LC433782.1
NSP4	RVA/Pig/China/LLP48/2008/G9P[6]	98.51%	E1	KJ126820.1
NSP5	RVA/Pig/CHN/SD-1/2021/G9P[23]	98.89%	H1	ON676179.1

### 3.4 Phylogenetic analysis of the full-length gene

**Figure 2:**
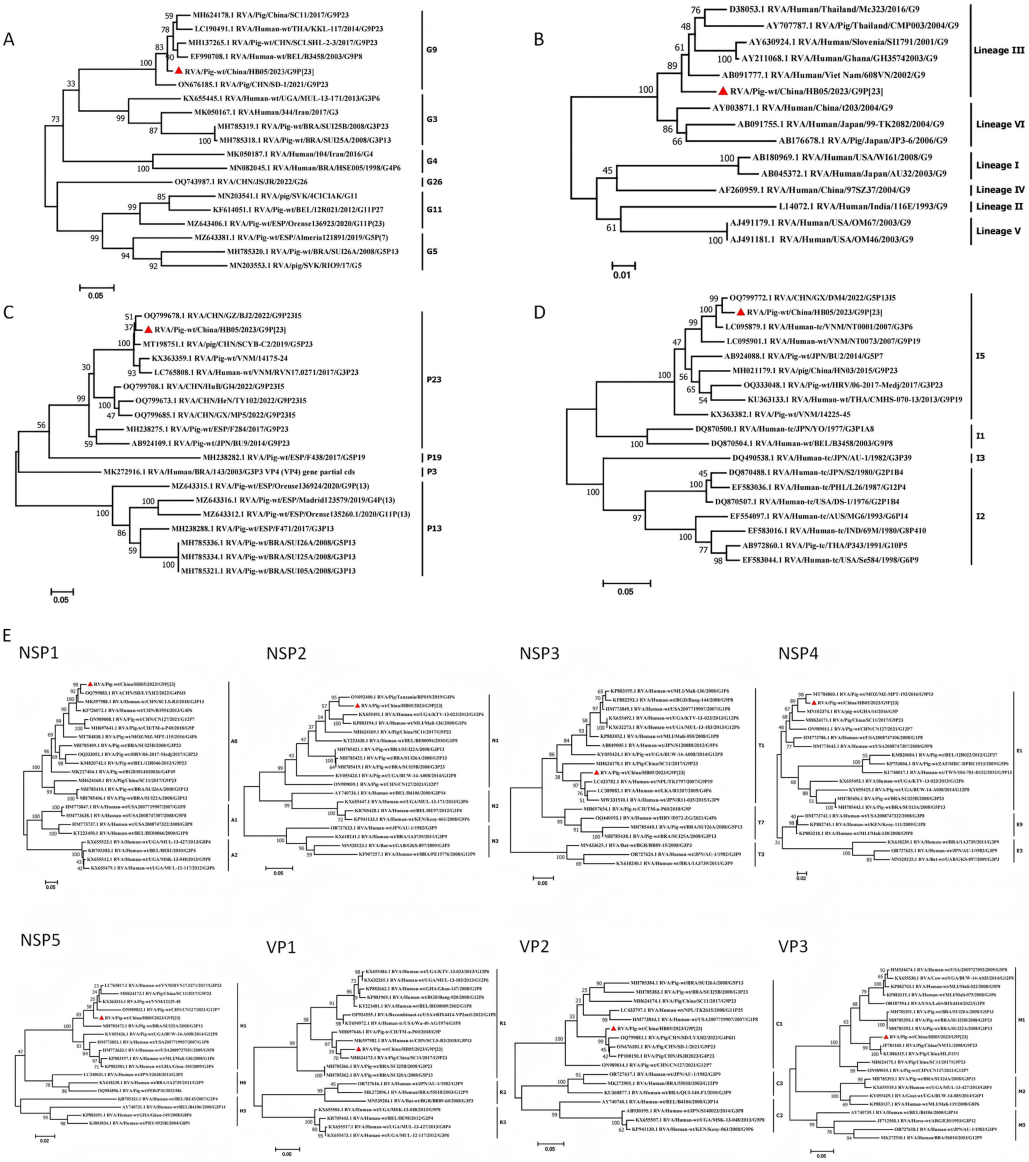
Phylogenetic analysis based on the nucleotide sequences of the 11 segments from HB05 and other strains by MEGA 7 using the maximum likelihood method. **(A)** Phylogenic trees based on VP7 gene from HB05 strain. **(B)** Phylogenetic analysis revealed that HB05 strain can be classified into the lineageIII. **(C)** Phylogenic trees based on VP4 gene from HB05 strain. **(D)** Phylogenic trees based on VP6 gene from HB05 strain. **(E)** Phylogenic trees based on the nucleotide of NSP1, NSP2, NSP3, NSP4, NSP5, VP1, VP2 and VP3 genes from HB05 strain.

Phylogenetic analysis revealed that the VP7 gene of HB05 strain clustered within lineage III of the G9 genotype. It showed a close genetic relationship with certain human RVA strains (Figure 2B). The HB05 strain shared a nucleotide sequence identity ranging from 85.4 to 93.7% with some human RVA strains belonging to lineage III within the G9 genotype (e.g., sharing 85.4% nucleotide sequence identity with Mc323). The VP7 gene segment was found to be grouped together with strains RVA/Pig/China/SC11/2017/G9P[23] and RVA/Pig/CHN/SD-1/2021/G9P[23] (Figure 2A). Moreover, phylogenetic analysis showed that the VP4 gene segment clustered within the same group as strain RVA/Pig-wt/CHN/GZ/BJ2/2022/G9P23I5, sharing a nucleotide sequence identity of 97.16% (Figure 2C). Additionally, when the VP6 nucleotide sequence was compared with the sequences reported in GenBank, it was found that strain HB05 belonged to branch I5 and had 98.24% nucleotide homology with CHN/GX/DM4/2022/G5P13I5 (Figure 2D). Collectively, these results suggest that the HB05 strain belonged to the porcine rotavirus A genotype G9P[23] I5, with its VP6, VP4, and VP7 gene segments originating from PoRV.

The VP1–VP3 genes of HB05 were, respectively, classified into genotypes R1, C1, and M1 (Figure 2E). The VP1 gene of HB05 strain clustered together with both porcine and human strains that had been identified in China. In contrast, the VP2 and VP3 segments of HB05 strain grouped together with porcine strains identified in China. The NSP segments of HB05 strain were assigned to the same group as the porcine strain RVA/Pig/China/SC11/2017/G9P[23]. Phylogenetic analysis revealed that the NSP1–NSP5 genes of HB05 strain were allocated to branches A8, N1, T1, E1, and H1, respectively (Figure 2E). The NSP1 segment clustered within the A8 clade. Given that this clade is predominantly composed of porcine isolates, it suggests that the NSP1 segment of the HB05 strain has a porcine origin. However, the NSP3 segment is likely of human origin, as the majority of isolates within this cluster are of human origin. The NSP2 segment of HB05 formed a distinct clade next to the RVA/pig/Tanzania/RP019/2019/G4P[6] strain (Figure 2). The NSP4 segment showed a high degree of similarity (98.51% nucleotide sequence identity) to the porcine strain RVA/Pig/China/LLP48/2008/G9P[6]. Meanwhile, the NSP5 segment exhibited a remarkable similarity (98.89% nucleotide sequence identity) to the Chinese porcine strain RVA/Pig/CHN/SD-1/2021/G9P[23] (Figure 2).

### 3.5 Comparison of VP4 and VP7 antigenic epitopes of the HB05 strain

**Figure 3:**
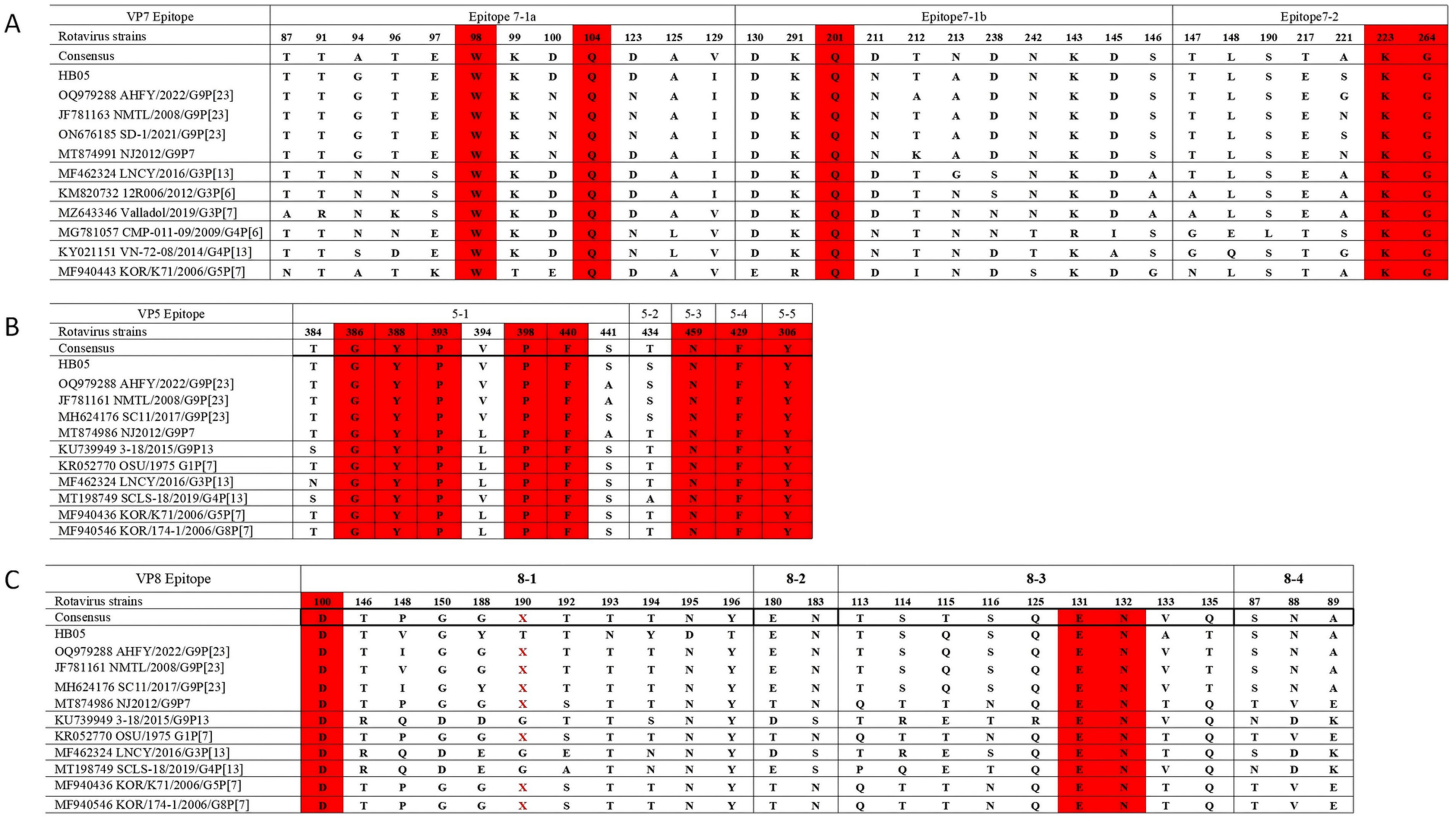
Comparison of VP4 (VP5* and VP8*) and VP7 antigenic epitopes in different strains. **(A)** Neutralizing epitopes on the VP7 protein. **(B)** Neutralizing epitopes on the VP5 protein. **(C)** Neutralizing epitopes on the VP8 protein.

The efficacy of vaccines may be compromised by alterations in the amino acid sequences of antigenic epitopes present on the VP7 trimer and VP4 multimer, which includes VP5* and VP8*. Dormitzer et al. (2002) and Aoki et al. (2009) elucidated the precise locations of these epitopes. When the VP4 spike protein undergoes proteolytic cleavage by trypsin, it generates two fragments, named VP8* (26 kDa) and VP5* (60 kDa). The VP5* subunit comprises five antigenic regions (5–1 to 5–5), whereas the VP8* subunit consists of four surface-exposed antigenic epitopes (8–1 to 8–4) (Figure 3). Potential trypsin cleavage sites in rotaviruses have been identified at positions 231, 241, 247, 467, and 582, which are characterized by the presence of conserved arginine residues across various strains of RVA. Additionally, a lysine residue at position 258 has been proposed as another potential trypsin cleavage site (Miao et al., 2022). Notably, the HB05 strain possesses all these conserved residues (data not presented).

The neutralizing epitope regions of the HB05-VP7 protein were compared with those of a series of RV strains retrieved from GenBank. Three amino acid positions (98 W, 104Q, and 201Q) in the 7–1 neutralizing epitope and two amino acid positions (223E and 264G) in the 7–2 neutralizing epitope were found to be conserved (Figure 3A). Four positions (100, 123, 212, 221) in the amino acid sequences of the 7–1 and 7–2 regions of HB05 strain differed from the RVA/Porcine/China/AHFY2022/2022/G9P[23] strain. There were also two different amino acid positions (100, 123) in the 7–1 and 7–2 sequences between the RVA/Pig/CHN/SD-1/2021/G9P[23] and HB05 strains. Three differences (100, 212, and 221) between the RVA/Porcine/China/NJ2012/2012/G9P[7] and HB05 strains were also observed in the amino acid sequences at positions 7–1 and 7–2. The neutralizing epitope region of VP5* from HB05 strain was specifically compared to a number of PoRV strains from GenBank. The results showed that, the neutralizing epitope of VP5* contains eight conserved amino acid residues (Y306, G386, Y388, P393, P398, F429, F440 and N459) except four variable amino acid residues (384, 394, 441, and 434) (Figure 3B). Among the 25 sites in the neutralizing epitope of VP8*, only three amino acids were conserved: one amino acid (100 D) in 8–1 and two amino acids (131, 132) in 8–3 (Figure 3C).

### 3.6 Pathogenicity in 3-day-old pig

**Figure 4:**
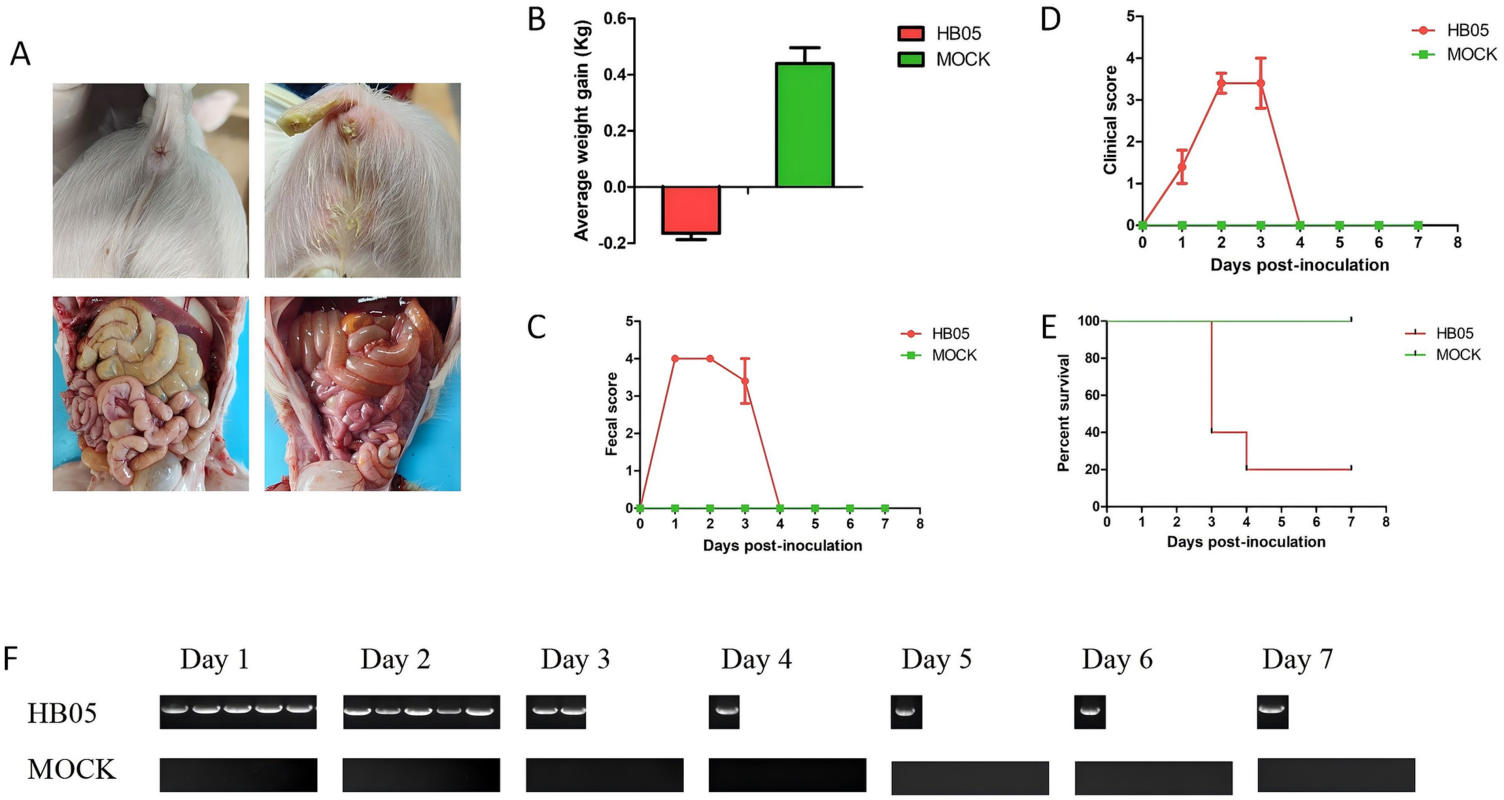
Pathogenicity of PoRV strain HB05 in 3-day-old piglets. 3-day-old piglets were orally inoculated with 2 mL HB05 virus and 2 mL DMEM (Control group) respectively (*n* = 5 in each group). **(A)** The Clinical signs and macroscopic lesions. **(B)** Average weight gain from days 0 to 7 post-challenged. **(C)** Fecal score was recorded each 24 h after inoculation until necropsy. **(D)** Clinical score was recorded each 24 h after inoculation until necropsy. **(E)** Viral shedding was detected using RT-PCR each 24 h after inoculation until necropsy.

To evaluate the pathogenicity of the HB05 strain, five 3-day-old piglets were orally inoculated with the HB05 (F9) virus at a dose of 5 × 10^5.5 TCID50/mL, while five control piglets were orally administration with DMEM. Compared with the control group, the challenged piglets showed marked diarrhea (Figure 4A). Post-mortem examination revealed macroscopic lesions in the challenged group, such as thin and translucent intestinal walls and intestines filled with yellowish watery fluid (Figure 4A). All five piglets in the HB05 group presented obvious dehydration and weight loss (Figure 4B). Throughout the experiment, the Control animals remained healthy without any clinical signs. Diarrhea was observed in all challenged piglets at 8 h post-infection (hpi), and by 24 hpi, all five had developed severe diarrhea (Figure 4C). The clinical symptoms reached their peak on the third day post-infection. Three piglets died on this day, and one more piglet died on the fourth day, leaving only one survivor (Figure 4E). This sole surviving piglet started to show alleviation of clinical symptoms on the third day and was free from diarrhea and other clinical symptoms by the fourth day, indicating a progressive recovery (Figure 4). Moreover, fecal viral shedding was monitored by RT-PCR targeting the PoRV VP7 gene. PoRV RNA was detected in all five rectal swab samples starting at 24 hpi, and viral shedding persisting until the piglet’s death (Figure 4F).

## 4 Discussion and conclusion

Recent studies have identified porcine rotavirus as a major contributor to diarrhea in piglets, with the prevalence of PoRVA in swine populations exhibiting substantial variability, ranging from 3.3 to 67.3%. Moreover, PoRVA infection has been demonstrated to elevate the transmission rates of other diarrhea-causing pathogens, such as PEDV and PDCoV (Jung et al., 2008; Li et al., 2022). In 2022, a comprehensive survey was conducted, collecting 8,588 pig samples from 2,075 farms across 29 provinces in China to assess the transmission dynamics of diarrhea-causing pathogens. The results indicated that 67.42% of the farms were positive for PoRVA, with a sample positivity rate of 44.00%. Interestingly, the prevalence of PoRVA surpassed that of PEDV, which had a sample positivity rate of 22.21% (Song et al., 2024). From 2021 to 2023, PoRVA emerged as the second most prevalent virus in southern China, with prevalence rates varying between 25.81 and 50.81%, and an overall farm prevalence rate of 72.77% (155/213) (Zhang et al., 2024). Additionally, the recent outbreak of severe diarrhea in piglets from March to August 2023 was attributed to Rotavirus A (RVA). Within these farms, various genotypes, including the historically common G genotypes G3, G4, G5, and G9, were identified notably, the majority of PoRVA strains belonged to the G9 subtype. Our findings are consistent with this, as genotype G9 accounted for approximately 58.16% (57/98) of the analyzed cases (Table 2). PoRVA G9 in combined with P[23], P[7] or P[13], constitutes the dominant genotype in the virus currently affecting diarrheic pigs (Monteagudo et al., 2022; Wu et al., 2017). The sequence analysis of porcine rotavirus type A (G9P[23]) in this study provides valuable insights into the prevention and control of its spread and is of great importance for future vaccine development against porcine rotavirus.

To establish an experimental foundation and provide technological insights into the etiological characteristics and molecular epidemiology of porcine rotavirus G9P[23] and other genotypes, we conducted pathogen detection on 98 diarrhea samples sourced from eight provinces. Our analysis revealed only PoRVA infections, and subsequent sequencing and alignment of the VP7 gene segments indicated that the strains and G genotypes of rotavirus affecting piglets varied across different provinces (Table 2). We isolated strains with identical VP7 sequences of the G9 genotype and performed high-throughput sequencing, revealing that infections in various regions, including Hebei, Liaoning, Sichuan, Zhejiang, and Henan, were caused by the same strain, which we have named HB05. The identification of this strain was confirmed using IFA and electron microscopy, confirming it as PoRV, and it was found to be capable of stable passage on MA104 cells (Figure 1). Next-generation sequencing (NGS) technology was employed to obtain the complete genome sequence of HB05 strain. The genome constellation of HB05 was found to be identical to previously identified Chinese porcine rotavirus G9P[23] strains, such as RVA/Pig/China/SC11/2017/G9P[23], which has the genotype G9-P[23]-I5-R1-C1-M1-A8-N1-T1-E1-H1 (Chen et al., 2019). However, all 11 genes of HB05 exhibited a relatively low level of sequence homology with strain RVA/Pig/China/SC11/2017/G9P[23] (Figure 2). Notably, the RVA/Pig/China/SC11/2017/G9P[23] strain was a human-porcine reassortment strain. The genetic analysis suggested that HB05 might have undergone further adaptation within the porcine population, possibly contributing to its increased prevalence in various regions. This raised concerns about the potential impact on the swine industry, emphasizing the need for ongoing surveillance and the development of effective vaccines to control the spread of this Porcine Rotavirus strain.

The complete genome of the HB05 G9P[23] strain has been sequenced and comprehensively analyzed, revealing its potential for cross-species transmission (Figure 2). The G9 genotype rotaviruses (RVAs) can be segregated into six distinct lineages (I–VI) based on the VP7 nucleotide sequence, with lineages I, II, IV, and V being exclusive to humans, and lineages III and VI being shared between humans and pigs (Esona et al., 2017; Phan et al., 2007; Shi et al., 2012). Nucleotide BLAST analysis of the VP7 gene segments revealed that the HB05 strain belongs to lineage III. Further BLAST analysis of individual gene segments indicated that the HB05 NSP3 gene is closely related to human G9 strains, exhibiting a 95.45% similarity to the strain RVA/Human-wt/NPL/TK1797/2007/G9P (Wang et al., 2018), and is distinct from other porcine G9 strain branches. In contrast, the VP1-VP4, VP6-VP7, NSP1-NSP2, and NSP4-NSP5 genes displayed the highest homology to porcine strains, with similarity levels ranging from 94.79 to 98.89%. These findings suggest that the HB05 strain may be a recombinant originating from both human and porcine sources. Among the 11 gene segments, seven (VP7, VP4, VP1, VP3, NSP3, NSP4, NSP5) exhibited the highest homology with the G9 genotype of rotavirus, three (VP2, NSP1, NSP2) with the G4 genotype of porcine rotavirus, and one (VP6) with the G5 genotype of porcine rotavirus (Table 3). This indicates that the HB05 strain may be a multi-genotype reassortant virus. Moreover, NSP2 exhibited the highest homology with the RVA/Pig/Tanzanian/RP019/2019/G4P6 strain, and NSP3 shared the highest homology with the RVA/Human-wt/NPL/TK1797/2007/G9P (Wang et al., 2018) strain, suggesting that the HB05 strain may be a reassortant derived from rotavirus strains from multiple countries. Although there is no direct evidence of rotavirus transmission between animals and humans, the reassortment of RVAs in animals suggests that these viruses have the potential for zoonotic transmission. Pigs are considered a potential host reservoir for human G9 RVAs (Matthijnssens et al., 2008). This underscores the ongoing mutation and evolution of the G9 strains, emphasizing the necessity for continuous surveillance of G9 rotaviruses.

Upon examining the genes encoding surface proteins, it has been determined that the G9 genotype possesses the capability to infect both humans and pigs, and is a prevalent VP7 genotype within porcine populations (Shi et al., 2012; Wang et al., 2024). Similarly, P[23] is also a significant genotype in pigs, predominantly occurring as part of the G9P[23] genotype combination in piglets. Genetic analysis of the HB05 genome has unveiled several unique mutations that may contribute to its adaptability and potential pathogenicity. The pathogenicity of strain HB05 was assessed by infecting 3-day-old piglets with PoRV (2 mL/piglet, 1.0 × 10^5.5 TCID50/ml). Diarrhea was observed at 8 h post-infection (hpi), and by 24 hpi, all pigs exhibited severe diarrhea and lethargy. By the fourth day post-infection, 4 out of 5 piglets had died to the infection, with only one surviving to the end of the experiment (Figure 4). However, a previous study indicated that 3-day-old piglets infected with the HN03 (G9P[23]) strain recovered after 72 h, despite experiencing severe watery diarrhea within the first 24 h (WANG et al., 2018). When 5-day-old piglets were challenged with RVA/Porcine/China/AHFY2022/2022/G9P[23] (2 mL/piglet, 1.0 × 10^6.5 TCID50/mL), 4 out of 5 of the challenged piglets exhibited mild-to-severe diarrhea at 36 hpi, and all piglets experienced diarrhea by 48 hpi (Shi et al., 2012). Notably, the duration of diarrhea was confined to one day, and importantly, none of the piglets succumbed to the infection, suggesting that our strain HB05 exhibits a higher pathogenicity compared to others. Further studies are warranted to elucidate the impact of these mutations on the virus’s interaction with the host’s immune system and its capacity to induce disease.

In conclusion, this study constitutes the first comprehensive characterization of a highly virulent human-porcine reassortant rotavirus A (RVA) G9P[23] strain, named HB05, isolated from nursing piglets experiencing severe diarrhea across multiple provinces in China. The findings highlight the necessity for vigilant surveillance to evaluate the prevalence, pathogenic mechanisms, and potential for interspecies transmission of the G9P[23] strain. It should be noted that the sample size was relatively small, comprising only 98 specimens randomly collected from pig farms in provinces with reported outbreaks of severe diarrhea. The samples we received were primarily from pig farms within our organization. Given the numerous similarities in the introduction of breeding pigs, feed supply, and personnel exchange among different pig farms within the same group, it is possible to observe 100% homology of the PoRV gene fragment between these farms. Nonetheless, the study offers significant insights into the virus’s characteristics. Given the limited geographical scope of this study, extrapolation of these findings to other regions of China should be approached with caution. Neutralization epitope analysis has uncovered unique mutations within the HB05 strain that may influence its adaptability and virulence. Further extensive research is imperative to clarify the implications of these mutations on the virus’s biological property. This comprehensive investigation, which encompasses whole-genome analysis and pathogenicity assessment of the HB05 rotavirus, enhances our comprehension of the evolutionary dynamics of G9P[23] strains and paves the way for the development of improved vaccines and control measures.

## Data Availability

The original contributions presented in the study are publicly available. This data can be found here: https://www.ncbi.nlm.nih.gov/genbank/, accession numbers PV212012-PV212022.
